# Highly Pathogenic Avian Influenza A(H5N1) Virus Clade 2.3.4.4b in Domestic Ducks, Indonesia, 2022

**DOI:** 10.3201/eid3003.230973

**Published:** 2024-03

**Authors:** Hendra Wibawa, Putut Eko Wibowo, Arif Supriyadi, Lestari Lestari, Jessiaman Silaban, Aziz Ahmad Fuadi, Anna Januar Fiqri, Retno Wulan Handayani, Sri Handayani Irianingsih, Zaza Fahmia, Herdiyanto Mulyawan, Syafrison Idris, Nuryani Zainuddin

**Affiliations:** Disease Investigation Center Wates, Yogyakarta, Indonesia (H. Wibawa, Lestari, J. Silaban, S.H. Irianingsih, Z. Fahmia, H. Mulyawan);; Disease Investigation Center Banjarbaru, Banjarbaru, Indonesia (P.E. Wibowo, A. Supriyadi, A.A. Fuadi, A.J. Fiqri, R.W. Handayani);; Directorate General of Livestock and Animal Health Services, Jakarta, Indonesia (S. Idris, N. Zainuddin)

**Keywords:** influenza, highly pathogenic avian influenza A, HPAI, H5N1, clade 2.3.4.4b, ducks, whole-genome sequencing, respiratory infections, viruses, zoonoses, Indonesia

## Abstract

Highly pathogenic avian influenza A(H5N1) clade 2.3.4.4b viruses were isolated from domestic ducks in South Kalimantan, Indonesia, during April 2022. The viruses were genetically similar to those detected in East Asia during 2021–2022. Molecular surveillance of wild birds is needed to detect potential pandemic threats from avian influenza virus.

The H5N1 subtype of the avian influenza virus A/goose/Guangdong/1/96 (Gs/GD/96) lineage has caused highly pathogenic avian influenza (HPAI) outbreaks in poultry since 1996. In 2008, various novel reassortant viruses were identified in domestic duck and live bird markets (LBMs) in China bearing the genetic backbone of Gs/GD/96 virus clade 2.3.4 hemagglutinin (HA) but different combinations of neuraminidase, such as H5N2, H5N5, H5N6, and H5N8 (*1*). Clade 2.3.4 continued to evolve into 5th order genetic groups (clades 2.3.4.4a–h); reassortment created different genotypes within those clades (*1*). H5N8 clade 2.3.4.4 viruses have predominantly spread across many countries in Asia to Europe, Africa, and North America (*1*,*2*); repeated outbreaks caused by H5N8 clade 2.3.4.4b viruses were reported during 2016 to mid-2020 (*3*,*4*). However, H5N1 clade 2.3.4.4b virus emerged in late 2020, which led to an increase in wild bird and poultry influenza outbreaks worldwide; this virus strain has almost entirely replaced H5N8 clade 2.3.4.4b globally since late 2021 (*5*). Moreover, the eastward movement of H5N1 clade 2.3.4.4b virus outbreaks from Europe to East Asia since late 2021 suggests that wild birds likely play a role in virus introduction (*5*,*6*).

## The Study

In April 2022, high numbers of poultry deaths were reported from 5 duck farms in Hulu Sungai Utara District, South Kalimantan Province, Indonesia (Appendix Figure 1). Approximately 4,430 of 5,770 (76.8%) ducks of different ages died; younger ducks manifested more severe disease. In July 2023, the deaths of 294 (135 adult and 159 young) of 450 ducks were reported in a Muscovy duck farm in Banjarbaru District of South Kalimantan Province. We collected oropharyngeal swab or tissue samples from ducks in Hulu Sungai Utara in 2022 and Banjarbaru in 2023 for necropsy and hematoxylin/eosin staining; gross and histologic pathology analyses were performed at the Disease Investigation Center Banjarbaru (Appendix). We also collected samples from ducks in LBMs within Banjar District (October 2022), which is located between the Hulu Sungai Utara and Banjarbaru districts where disease was reported (Appendix Figure 1). We sent all influenza A(H5) PCR–positive samples to the Disease Investigation Center Wates in Yogyakarta, where viruses were isolated by using the World Organisation for Animal Health protocol (*7*). However, viruses could only be isolated from 3 pooled swab samples from the initial cases in April 2022 in Hulu Sungai Utara, 1 tissue sample from the July 2023 case in Banjarbaru, and 1 pooled swab sample from LBMs in Banjar. We characterized the virus isolates antigenically by using hemagglutination inhibition assays and genetically by using whole-genome sequencing on an Illumina sequencing platform (https://www.illumina.com) (Appendix).

We deposited whole-genome sequences of 4 virus isolates into the GISAID database (https://www.gisaid.org) under accession nos. EPI_ISL_17371282 (A/duck/Hulu Sungai Utara/A0522064-06/2022), EPI_ISL_17371283 (A/duck/Hulu Sungai Utara/A0522064-03-04/2022), EPI_ISL_17371284 (A/duck/Hulu Sungai Utara/A0522067-06-07/2022), and EPI_ISL_18438033 (A/Muscovy duck/Banjarbaru/A0523532-9/2023). All 5 identified virus isolates were H5N1 clade 2.3.4.4b viruses, but the virus isolate from LBMs in Banjar District was not included in further analysis or deposited in the GISAID database because of incomplete gene sequences (<50% full-length sequence for each gene segment).

Phylogenetic analysis of the HA gene segment showed that all 4 analyzed viruses clustered with recent HPAI H5 clade 2.3.3.4b viruses from Asia and Europe (Figure). However, they appeared to be more closely related to H5N1 clade 2.3.4.4b viruses from wild birds and poultry from Japan, China, and South Korea isolated during October 2021–February 2022. Phylogenetic trees for the other gene segments (polymerase basic 1, polymerase basic 2, polymerase acidic, nucleoprotein, neuraminidase, matrix protein, and nonstructural segments) also indicated that all 4 viruses were closely related to H5N1 clade 2.3.4.4b from Japan, China, and South Korea (Appendix Figures 2–5). The 3 viruses isolated from the influenza outbreak in April 2022 shared 99.8%–100% nucleotide sequence similarity for each viral segment; however, we observed a lower nucleotide sequence similarity between the viruses from April 2022 and the virus isolated in July 2023 (Table 1), indicating that H5N1 clade 2.3.4.4b continued to mutate resulting in genetic drift. We identified all virus isolates as HPAI on the basis of amino acid sequences within the HA cleavage site (REKRRKR|G); none of those isolates had molecular determinants associated with increased binding affinity or replication efficiency in mammals, including humans (Appendix Table 1) (*8*,*9*). A BLAST search (https://www.ncbi.nlm.nih.gov/blast) and pairwise distance analysis indicated all 8 gene segments from viruses isolated during the first outbreak in April 2022 had 98.4%–99.8% nucleic acid sequence identities to H5N1 clade 2.3.4.4b viruses from Japan, China, and South Korea, suggesting a close common ancestor.

**Figure:**
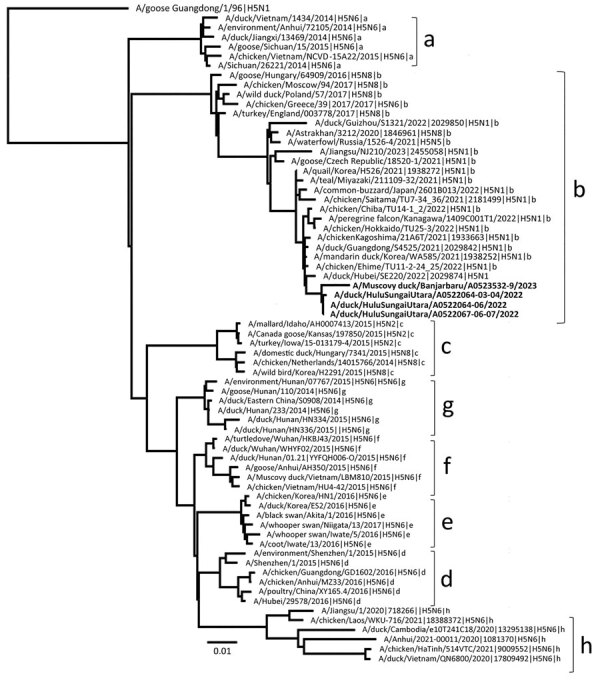
Phylogenetic analysis of the hemagglutinin gene of highly pathogenic avian influenza A(H5N1) clade 2.3.4.4b viruses isolated from domestic ducks during outbreaks in South Kalimantan, Indonesia, in April 2022 and July 2023 compared with reference sequences. Bold font indicates the viruses isolated from duck farms in this study. Letters at right indicate subclades. Evolutionary history was inferred by using the maximum-likelihood method and best-fit general time reversible plus gamma distribution 4 substitution model involving 67 hemagglutinin H5 sequences from the GISAID database (http://www.gisaid.org); a total of 1,656 positions were in the final dataset. Scale bar indicates nucleotide substitutions per site.

**Table 1:** DNA sequence homologies between highly pathogenic avian influenza A(H5N1) clade 2.3.4.4b viruses isolated from domestic ducks in Indonesia, 2022, and those from Banjarbaru and East Asia*

Virus name	GISAID no.†	Collection date	% Nucleic acid similarity for each gene segment							
			PB2	PB1	PA	HA	NP	NA	MP	NS
Viruses from first outbreak in Hulu Sungai Utara	EPI_ISL_17371282, EPI_ISL_17371283, EPI_ISL_17371284	2022 Apr	100	100	99.8–99.9	99.9–100	100	100	100	99.8–100
A/Muscovy duck/Banjarbaru/A0523532-9/2023	EPI_ISL_18438033	2023 Jul 7	99.4	99.3–99.4	99.1–99.2	98.9–99.0	99.1	98.8	99.8	98.7
A/mandarin duck/Korea/WA585/2021	EPI_ISL_6959592	2021 Oct 26	99.6	99.5–99.6	99.6–99.7	99.2–99.3	99.6	99.8	99.6	99.4
A/quail/Korea/H526/2021	EPI_ISL_6959593	2021 Nov 8	99.4	99.2	99.2–99.3	99.0–99.1	99.3	99.6	99.3	99.2
A/duck/Guangdong/S4525/2021	EPI_ISL_12572655	2021 Dec 8	99.6	99.6	99.3–99.4	99.2–99.3	99.5	99.7	99.7	99.2
A/duck/Hubei/SE220/2022	EPI_ISL_12572659	2022 Jan 10	99.6	99.5	99.3–99.4	99.0–99.2	99.5	99.5	99.6	99.2
A/duck/Guizhou/S1321/2022	EPI_ISL_12572656	2022 Feb 22	99.6	99.6	99.5–99.6	97.2–97.3	99.5	99.4	99.8	99.2
A/chicken/Kagoshima/21A6T/2021	EPI_ISL_6829533	2021 Nov 12	99.6	99.6	99.6–99.7	99.1–99.2	99.6	99.7	99.8	99.4
A/chicken/Saitama/TU7-34,36/2021	EPI_ISL_15063425	2021 Dec 7	99.6	99.3–99.4	99.1–99.2	98.4–98.6	99.1	99.6	99.3	99.0
A/chicken/Ehime/TU11-2-24 25/2022	EPI_ISL_15063431	2022 Jan 4	99.8	92.0	99.5–99.6	99.2–99.3	99.3	99.6	99.7	99.3
A/common buzzard/Japan/2601B013/2022	EPI_ISL_16831015	2022 Jan 27	99.6	99.2–99.3	99.3–99.4	98.6–98.7	99.3	99.6	99.3	99.2
A/teal/Miyazaki/211109-32/2021	EPI_ISL_15613494	2021 Nov 9	99.4	99.2–99.3	99.3	98.7–98.8	99.1	99.6	99.2	99.2

*H5N1 clade 2.3.4.4b viruses isolated from the initial poultry outbreak in Hulu Sungai Utara in April 2022 were compared with those isolated later from Banjarbaru, Indonesia, in July 2023 and H5N1 clade 2.3.4.b viruses from East Asia isolated during October 2021–February 2022. HA, hemagglutinin; MP, matrix protein; NA, neuraminidase; NP, nucleoprotein; NS, nonstructural; PA, polymerase acidic; PB1, polymerase basic 1; PB2, polymerase basic 2. †GISAID database (https://www.gisaid.org).

The gross and histologic pathology of naturally infected ducks showed multiorgan hemorrhages with prominent lesions in tissues and congestion and focal necrosis in parenchymal cells, often accompanied by inflammatory cell infiltrates (Appendix, Figure 6). Hemagglutination inhibition assays revealed the virus isolates from April 2022 had low reactivity with H5N1 antiserum derived from circulating viruses, including the H5N1 vaccine strains used for poultry (Table 2). Those results suggest that new vaccine candidates antigenically matched to circulating viruses might be needed in Indonesia, if H5N1 clade 2.3.4.4b viruses continue to infect poultry.

**Table 2:** Hemagglutinin inhibition assay titers using 2-fold serial dilutions of virus-specific antiserum in study of highly pathogenic avian influenza A(H5N1) virus clade 2.3.4.4b in domestic ducks, Indonesia, 2022*

Antiserum source, clade, GISAID no.†	Antigen source			
	A/duck/Hulu Sungai Utara/A0522064-06/2022	A/duck/Hulu Sungai Utara/A0522064-03-04/2022	A/duck/Hulu Sungai Utara/A0522067-06-07/2022	A/muscovy duck/ Banjarbaru/A0523532-9/2023
A/chicken/West Java/PWT-WIJ/2006, H5N1 clade 2.1.3.2, EPI_ISL_12700530‡	<4	<4	<4	16
A/chicken/Barru/BBVM 41-13/2013, H5N1 clade 2.1.3.2a, EPI_ISL_17767706	16	16	16	16
A /duck/Sukoharjo/BBVW-1428-9/2012, H5N1 clade 2.3.2.1c, EPI_ISL_266808§	16	32	32	32
A/chicken/Tanggamus/031711076-65/2017, H5N1 clade 2.3.2.1c, EPI_ISL_17767763¶	16	32	16	32
A/duck/Laos/XBY004/2014, H5N6 clade 2.3.4.4b, EPI_ISL_168385	8	16	8	16
A/duck/Hulu Sungai Utara/A0522064-03-04/2022, H5N1 clade 2.3.4.4b#	512	512	128	128

*Viruses were isolated by using the World Organisation for Animal Health protocol (*7*). The 3 viruses isolated from Hulu Sungai Utara in April 2022 and the virus isolated from Banjarbaru in July 2023 were used as antigen sources. †GISAID (https://www.gisaid.org). ‡H5N1 clade 2.1.3.2 vaccine-seed strain used in Indonesia since 2009. §H5N1 clade 2.3.2.1c vaccine strain used during 2012–2020. ¶H5N1 clade 2.3.2.1c vaccine strain used since 2021. #Homologous antiserum for H5N1 clade 2.3.4.4b viruses isolated from the initial outbreak in Hulu Sungai Utara, April 2022.

Wild migratory birds might play a role in the intercontinental spread of HPAI H5Nx clade 2.3.4.4 viruses (*1*,*10*,*11*). Indonesia is situated within the East Asian Flyway’s island or oceanic routes linking eastern Russia and Japan to the Philippines and eastern Indonesia (*12*). One stopover site is on the west coast of South Kalimantan, where 23 migratory bird species have been identified and observed (*13*). Migratory birds often use stopover sites for 1 day to several weeks to rest and refuel (*12*), providing opportunities for virus transmission through direct or indirect contacts with local wild birds or aquatic poultry within their shared habitats.

During April 2022–July 2023, we conducted molecular surveillance through a network for influenza virus monitoring in Indonesia (*14*) and did not detect other H5N1 clade 2.3.4.4b outbreaks outside of South Kalimantan. Similar to an earlier virus incursion of H5N1 clade 2.3.2.1c in Java in 2012, which initially also affected ducks (*15*), we could not determine the exact origin of virus incursion. However, genetic evidence and bird migration patterns suggest that migratory birds contributed to the introduction of H5N1 clade 2.3.4.4b into Indonesia.

## Conclusion

We identified HPAI H5N1 clade 2.3.4.4b viruses in ducks in South Kalimantan, Indonesia. The role of migratory birds in virus introduction cannot be ruled out because South Kalimantan is situated within the East Asia Flyway corridor, and the infected farms were connected to marshes that provided opportunity for direct or indirect contacts with migratory birds. Limited wild bird surveillance and genome sequence data for avian influenza viruses impeded our ability to determine further transmission and spread of H5N1 clade 2.3.4.4b in Indonesia. Both epidemiologic studies and molecular surveillance of wild birds are needed to better prepare for pandemic threats caused by continued avian influenza virus evolution in Indonesia and elsewhere.

Appendix Additional information for highly pathogenic avian influenza A(H5N1) virus clade 2.3.4.4b in domestic ducks, Indonesia, 2022.
