# Supplementary material for: Highly Pathogenic Avian Influenza A(H5N1) Virus Clade 2.3.4.4b in Domestic Ducks, Indonesia, 2022
Source: Emerg Infect Dis. 2024 Mar;30(3):586–90. doi: 10.3201/eid3003.230973 (PMC10902521; doi:10.3201/eid3003.230973)
Supplement: Appendix — Additional information for highly pathogenic avian influenza A(H5N1) virus clade 2.3.4.4b in domestic ducks, Indonesia, 2022. [file 23-0973-Techapp-s1.pdf]

Article DOI: https://doi.org/10.3201/eid3003.230973

EID cannot ensure accessibility for supplementary materials supplied by authors. Readers who have difficulty accessing supplementary content should contact the authors for assistance.

Highly Pathogenic Avian Influenza A(H5N1) Virus Clade 2.3.4.4b in Domestic Ducks, Indonesia, 2022

Appendix

Material and Methods

Samples

In April 2022, we collected 18 oropharyngeal swab and 2 tissue samples from ducks within 3 of 5 duck farms located close to marshes in 2 villages (Sungai Malang and Mamar) in Hulu Sungai Utara District, South Kalimantan Province, Indonesia, that reported duck deaths with neurologic signs. In addition, 35 oropharyngeal swab and 7 tissues samples were collected from domestic ducks for sale in live bird markets (LBMs) in Banjar District in October 2022; 8 tissue and 5 oropharyngeal swab samples were also collected from ducks at a Muscovy duck farm in Banjarbaru Distict in July 2023. Oropharyngeal swab samples were pooled; pooled samples comprised 4–5 swab samples that were placed into viral transfer media in the field. Those samples were collected by the Disease Investigation Center (DIC) Banjarbaru; samples that were influenza A H5–positive by PCR were sent to the National Avian Influenza Reference Laboratory at DIC Wates for virus isolation in 9–10-day-old specific pathogen-free chicken embryonic eggs by using the WOAH protocol (*1*). Viruses could be isolated from only 3 pooled swab samples from the initial duck cases in April 2022 in Hulu Sungai Utara, 1 pooled swab sample from an LBM in Banjar, and 1 tissue sample from July 2023 in Banjarbaru. Viruses were characterized antigenically by using hemagglutination inhibition assays and genetically by whole-genome sequencing for avian influenza virus.

Whole-Genome Sequencing and Phylogenetic Analysis

We performed RNA extraction by using the QIAamp Viral RNA Mini Kit (Qiagen, https://www.qiagen.com) and multisegment reverse transcription PCR with specific primers, MBTuni-12 and MBTuni-13, to amplify all 8 gene segments of avian influenza virus (*2*). DNA libraries were prepared by using the Nextera-XT DNA Library Preparation Kit (Illumina, https://www.illumina.com) according to the manufacturer`s instructions. Whole-genome sequencing was performed by using the MiSeq next-generation sequencing instrument and MiSeq Reagent Kit v3 (both Illumina). Validation and assembly of nucleotide sequences were performed by using Geneious Prime version 2022.2.1 (Geneious, https://www.geneious.com).

Complete genome sequences of A/duck/Hulu Sungai Utara/A0522064–06/2022 (GISAID accession no. EPI_ISL_17371282), A/duck/Hulu Sungai Utara/A0522064–03–04/2022 (no. EPI_ISL_17371283), A/duck/Hulu Sungai Utara/A0522067–06–07/2022 (no. EPI_ISL_17371284), and A/Muscovy duck/ Banjarbaru/A0523532–9/2023 (no. EPI_ISL_18438033) have been deposited in the GISAID database (https://www.gisaid.org). The virus isolate from 1 duck sampled in an LBM in Banjar (A/duck/Banjar/A0522477–74/2022) was identified as avian influenza A(H5N1) clade 2.3.4.4b, but was not included in downstream analyses because of incomplete sequences (partial genes, <50% full-length sequences for each segment). The PhyML Maximum Likelihood method in Unipro UGENE v.46 (*3*) was used for phylogenetic analysis of each gene segment; the general time-reversible nucleotide substitution model with 4 discrete gamma categories was used. Final dendrograms of Newick trees were generated and visualized in FigTree v.1.4.4 (https://github.com/rambaut/figtree/releases). We performed BLAST tool searches (https://www.ncbi.nlm.nih.gov/blast) and nucleotide identity analyses, which calculated identities from the output of pairwise distance analysis for each gene segment in MEGA X (*4*).

Hemagglutination Inhibition Test

We performed hemagglutination inhibition assays by using the WOAH standard method (*1*) to test the reactivity of 3 virus isolates against representative antiserum derived from H5N1 clade 2.1.3.2 virus strains (very few still detected in poultry), clade 2.3.2.1c virus strains (dominant circulating virus clade in poultry), H5N6 clade 2.3.4.4b (A/duck/Laos/XBY004/2014), and the homologous H5N1 clade 2.3.4.4b strain (A/duck/Hulu Sungai Utara/A0522064–03–04/2022).

Gross and Histologic Pathology

Carcasses from dead ducks were necropsied by veterinary pathologists within the postmortem facility in DIC Banjarbaru. Tissue samples included brain, lungs, heart, liver, spleen, pancreas, intestines, and kidney and were processed and embedded into wax by using routine histologic laboratory processes. Embedded tissues were sliced by using standard microtomy methods, and consecutive 4-µm-thick sections were stained with hematoxylin and eosin.

References

1. World Organization for Animal Health. WOAH terrestrial manual 2021, Chapter 3.3.4. Avian influenza (including infection with high pathogenicity avian influenza viruses) [cited 2023 Jan 2]. https://www.woah.org/fileadmin/Home/eng/Health_standards/tahm/3.03.04_AI

2. Zhou B, Donnelly ME, Scholes DT, St George K, Hatta M, Kawaoka Y, et al. Single-reaction genomic amplification accelerates sequencing and vaccine production for classical and swine origin human influenza a viruses. J Virol. 2009;83:10309–13. PubMed https://doi.org/10.1128/JVI.01109-09

3. Okonechnikov K, Golosova O, Fursov M; UGENE team. Unipro UGENE: a unified bioinformatics toolkit. Bioinformatics. 2012;28:1166–7. PubMed https://doi.org/10.1093/bioinformatics/bts091

4. Kumar S, Stecher G, Li M, Knyaz C, Tamura K. MEGA X: molecular evolutionary genetics analysis across computing platforms. Mol Biol Evol. 2018;35:1547–9. PubMed https://doi.org/10.1093/molbev/msy096

Appendix Table 1. Amino acid changes in protein segments from highly pathogenic avian influenza A(H5N1) viruses related to increased binding activity and replication in mammal cells or increased virulence in mammals in study of outbreak in domestic ducks, Indonesia, 2022*

	Virus protein segments																
	PB2			PB1	PB1-F2	PA	HA					NA	M2	NS		
Virus, clade	Q591K	E627K	D701N	N105S	N66S	T515I	Cleavage site	Q192R	Q222L	S223N	G224S	Stalk deletion	S31N	Deletion aa 80−84	P42S	PDZ motif
A/Vietnam/1203/2004, clade 3A.1	Q	K	D	N	N	T	RERRRKKR|G	Q	Q	S	G	Yes	N	Yes	S	ESEV
A/Jiangsu/NJ210/2023, clade 2.3.3.4b	Q	E	D	N	N	T	REKRRKR|G	K	Q	R	G	No	S	No	S	ESEV
A/duck/Hulu Sungai Utara/A0522064-06/2022	Q	E	D	N	N	T	REKRRKR|G	K	Q	R	G	No	S	No	S	ESEV
A/duck/Hulu Sungai Utara/A0522064-03-04/2022	Q	E	D	N	N	T	REKRRKR|G	K	Q	R	G	No	S	No	S	ESEV
A/duck/Hulu Sungai Utara/A0522067-06-07/2022	Q	E	D	N	N	T	REKRRKR|G	K	Q	R	G	No	S	No	S	ESEV
A/muscovy duck/Banjarbaru/A0523532-9/2023	Q	E	D	N	N	T	REKRRKR|G	K	Q	R	G	No	S	No	S	EPEV

*HA, hemagglutinin; M2, matrix protein 2; NA, neuraminidase; NS, nonstructural; PA, polymerase acidic; PB1, polymerase basic 1; PB1-F2, polymerase basic 1-frame 2; PB2, polymerase basic 2; PDZ, postsynaptic density protein-95/discs large/zonula occludens-1.

Appendix Table 2. Sequence acknowledgment table for using GISAID EpiFlu database*

No.	Isolate name	GISAID no.	Country	Collection date	Originating Laboratory	Submitting Laboratory	Authors
1	A/Goose/Guangdong/1/96	EPI_ISL_1254	China	1996-Jan-01	NA	Import from public domain	NA
2	A/duck/Vietnam/1434/2014	EPI_ISL_177703	Vietnam	2014-Nov-18	NA	Import from public domain	Hatamachi, J.; Ogaswara, K.; Chu, D.H.; Okamatsu, M.; Sakoda, Y.; Kida, H.; Ogasawara, K.
3	A/Environment/Anhui/72105/2014	EPI_ISL_219801	China	2014-May-20	NA	WHO Chinese National Influenza Center	Wang, Dayan; Li, Xiaodan; Zou, Shumei; Zhang, Ye; Bo, Hong; Li, Xiyan; Chen, Wenbing; Yang, Lei; Shu, Yuelong
4	A/duck/Jiangxi/13469/2014	EPI_ISL_173478	China	2014-Mar-30	NA	Import from public domain	Ma, C.; Lam, T.T.Y.; Chai, Y.; Wang, J.; Fan, X.; Hong, W.; Zhang, Y.; Li, L.; Liu, Y.; Smith, D.K.; Webby, R.J.; Peiris, J.S.M.; Zhu, H.; Guan, Y.
5	A/chicken/Yangzhou/YD1/2014	EPI_ISL_295144	China	2014-Sep-01	NA	Import from public domain	Li, J.; Gu, M.; Sun, W.; Liu, K.; Gao, R.; Liu, D.; Hu, J.; Wang, X.; Hu, S.; Liu, X.

No.	Isolate name	GISAID no.	Country	Collection date	Originating Laboratory	Submitting Laboratory	Authors
6	A/Sichuan/26221/2014	EPI_ISL_163493	China	2014-Apr-21	NA	WHO Chinese National Influenza Center	NA
7	A/chicken/Vietnam/NCVD-15A22/2015	EPI_ISL_244487	Vietnam	2015-Apr-02	NA	Import from public domain	Davis, T.; Jang, Y.
8	A/goose/SiChuan/15/2015	EPI_ISL_255850	China	2015-Apr-07	NA	Wuhan Institute of Virology	NA
9	A/Goose/Hungary/64909/2016	EPI_ISL_271713	Hungary	2016-Dec-14	National Food Chain Safety Office Veterinary Diagnostic Directorate Laboratory for Molecular Biology	DaNAm.Vet. Molbiol	Adam, Dan
10	A/chicken/Moscow/94/2017	EPI_ISL_17767843	Russian Federation	2017-Feb-28	N.F. Gamaleya Research Center for Epidemiology and Microbiology	Import from public domain	Voronina, O.L.; Ryzhova, N.N.; Aksenova, E.I.; Kunda, M.S.; Sharapova, N.A.N.E.; Fedyakina, I.T.; Chvala, I.A.; Borisevich, S.V.; Loguniv, D.Y.; Gintsburg, A.L.
11	A/wild duck/Poland/57/2017	EPI_ISL_300745	Poland	2017-Jan-27	NA	National Veterinary Research Institut Poland, PIWet-PIB	Swieton, E.; Smietanka, K.
12	A/chicken/Greece/39_2017/2017	EPI_ISL_288362	Greece	2017-Feb-06	Thessalonica Veterinary Centre (TVC)	Animal and Plant Health Agency (APHA)	Seekings, James; Ellis, Richard; Brookes, Sharon M.; Reid, Stephen; Lewis, Nicola; Brown, Ian H.; Dovas, C.; Georgiades, D.
13	A/turkey/England/003778/2017	EPI_ISL_253036	United Kingdom	2017-Jan-15	Animal and Plant Health Agency (APHA)	Animal and Plant Health Agency (APHA)	Seekings, James; Ellis, Richard; Brookes, Sharon M.; Reid, Scott; Essen, Stephen; Brown, Ian H.
14	A/duck/Guizhou/S1321/2022	EPI_ISL_12572656	China	2022-Feb-22	Harbin Veterinary Research Institute (CAAS)	Harbin Veterinary Research Institute (CAAS)	Pengfei Cui; Congcong Wang
15	A/Astrakhan/3212/2020	EPI_ISL_1038924	Russian Federation	2020-Dec-12	Center of Hygiene and Epidemiology in Astrakhan Region	State Research Center of Virology and Biotechnology (VECTOR)	Pyankova, O.; Susloparov, I.; Marchenko, V.; Ryzhikov, A.
16	A/goose/Czech Republic/18520-1/2021	EPI_ISL_17767177	Czech Republic	2021-Sep-27	State Veterinary Institute Prague	Import from public domain	Nagy, A.; Cernikova, L.; Stara, M.
17	A/chicken/Saitama/TU7-34,36/2021	EPI_ISL_15063425	Japan	2021-Dec-07	NA	Import from public domain	Soda, K.; Usui, T.; Ito, H.; Yamaguchi, T.; Ito, T.
18	A/teal/Miyazaki/211109-32/2021	EPI_ISL_15613494	Japan	2021-Nov-09	NA	Import from public domain	Soda, K.; Mekata, H.; Yamada, K.; Ito, H.; Usui, T.; Yamaguchi, T.; Ito,T.
19	A/common buzzard/Japan/ 2601B013/2022	EPI_ISL_16831015	Japan	2022-Jan-27	NA	Import from public domain	Soda, K.; Ito, H.; Usui, T.; Yamaguchi, T.; Ito, T.

No.	Isolate name	GISAID no.	Country	Collection date	Originating Laboratory	Submitting Laboratory	Authors
20	A/quail/Korea/H526/2021	EPI_ISL_6959593	Korea, Republic of	2021-Nov-08	Animal and Plant Quarantine Agency (O-2144)	Animal and Plant Quarantine Agency (APQA)	NA
21	A/peregrine falcon/Kanagawa/ 1409C001T1/2022	EPI_ISL_15923322	Japan	2022-Sep-25	National Institute for Environmental Studies	National Institute of Animal Health	Manabu, Onuma; Kei, Nabeshima; Atsushi, Haga; Hisako, Honjo; Misako, Yokoyama; Yuko, Uchida; Kohtaro, Miyazawa; Ryota, Tsunekuni; Junki, Mine; Saki,Sakuma; Asuka, Kumagai; Yoshihiro Takadate
22	A/chicken/Ehime/TU11-2-24,25/2022	EPI_ISL_15063431	Japan	2022-Jan-04	NA	Import from public domain	Soda, K.; Ito, H.; Hisada, R.; Usui, T.; Yamaguchi, T.; Ito, T.
23	A/chicken/Kagoshima/21A6T/2021	EPI_ISL_6829533	Japan	2021-Nov-12	National Institute of Animal Health	National Institute of Animal Health	NA
24	A/mandarin duck/Korea/WA585/2021	EPI_ISL_6959592	Korea, Republic of	2021-Oct-26	Animal and Plant Quarantine Agency (O-2144)	Animal and Plant Quarantine Agency (APQA)	NA
25	A/duck/Guangdong/S4525/2021	EPI_ISL_12572655	China	2021-Dec-08	Harbin Veterinary Research Institute (CAAS)	Harbin Veterinary Research Institute (CAAS)	Pengfei Cui; Congcong Wang
26	A/duck/Hubei/SE220/2022	EPI_ISL_12572659	China	2022-Jan-10	Harbin Veterinary Research Institute (CAAS)	Harbin Veterinary Research Institute (CAAS)	Pengfei Cui; Congcong Wang
27	A/duck/Hulu Sungai Utara/A0522064-06/2022	EPI_ISL_17371282	Indonesia	2022-Apr-04	Disease Investigation Centre Regional V Banjarbaru (BPPVRV)	Balai Besar Veteriner Wates	Wibawa, Hendra; Wibowo, Putut Eko; Lestari; Irianingsih, Sri Handayani; Supriyadi, Arif; Fiqri, Anna Januar; Fahmia, Zaza; Silaban, Jesiaman; Mulyawan, Herdiyanto
28	A/duck/Hulu Sungai Utara/A0522064-03-04/2022	EPI_ISL_17371283	Indonesia	2022-Apr-04	Disease Investigation Centre Regional V Banjarbaru (BPPVRV)	Balai Besar Veteriner Wates	Wibawa, Hendra; Wibowo, Putut Eko; Lestari; Irianingsih, Sri Handayani; Supriyadi, Arif; Fiqri, Anna Januar; Fahmia, Zaza; Silaban, Jesiaman; Mulyawan, Herdiyanto
29	A/duck/Hulu Sungai Utara/A0522067-06-07/2022	EPI_ISL_17371284	Indonesia	2022-Apr-04	Disease Investigation Centre Regional V Banjarbaru (BPPVRV)	Balai Besar Veteriner Wates	Wibawa, Hendra; Wibowo, Putut Eko; Lestari; Irianingsih, Sri Handayani; Supriyadi, Arif; Fiqri, Anna Januar; Fahmia, Zaza; Silaban, Jesiaman; Mulyawan, Herdiyanto
30	A/muscovy duck/Banjarbaru/ A0523532-9/2023	EPI_ISL_18438033	Indonesia	2022-Apr-04	Disease Investigation Centre Regional V Banjarbaru (BPPVRV)	Balai Besar Veteriner Wates	Wibawa, Hendra; Wibowo, Putut Eko; Lestari; Irianingsih, Sri Handayani; Supriyadi, Arif; Fiqri, Anna Januar; Fahmia, Zaza; Silaban, Jesiaman; Mulyawan, Herdiyanto
31	A/domestic duck/Hungary/7341/2015	EPI_ISL_177584	Hungary	2015-Feb-23	Danam.Vet.Molbiol	Danam.Vet. Molbiol	Krisztian, Banyai; Szilvia, Farkas; Adam, Dan
32	A/chicken/Netherlands/14015766/2014	EPI_ISL_174349	Netherlands	2014-Nov-19	Wageningen Bioveterinary Research	Wageningen Bioveterinary Research	Heutink, Rene; Harders, Frank; Verschuren-Pritz, Sylvia; Bossers, Alex; Koch, Guus; Bouwstra, Ruth

No.	Isolate name	GISAID no.	Country	Collection date	Originating Laboratory	Submitting Laboratory	Authors
33	A/wild bird/Korea/H2291/2015	EPI_ISL_234336	Korea, Republic of	2015-Jan-30	NA	Animal and Plant Quarantine Agency (APQA)	NA
34	A/mallard/Idaho/AH0007413/2015	EPI_ISL_206408	United States	2015-Jan-17	NA	Import from public domain	Killian, M.L.
35	A/Canada goose/Kansas/197850/2015	EPI_ISL_206450	United States	2015-Mar-13	NA	Import from public domain	Killian, M.L.; Ip, H.S.; Griffin, K.; Messer, J.; McMullen, K.; Long, R.; Hesting, S.
36	A/turkey/Iowa/15-013179-4/2015	EPI_ISL_301110	United States	2015-Jan-01	NA	Import from public domain	Lee, D.-H.; Torchetti, M.; Hicks, J.; Killian, M.; Bahl, J.; Pantin-Jackwood, M.; Swayne, D.
37	A/Environment/Shenzhen/1/2015	EPI_ISL_205314	China	2015-Dec-21	NA	WHO Chinese National Influenza Center	Fang, Shisong; Yang, Lei
38	A/Shenzhen/1/2015	EPI_ISL_205313	China	2015-Dec-28	Shenzhen center for disease control and prevention	WHO Chinese National Influenza Center	Fang, Shisong; Yang, Lei
39	A/poultry/China/XY165.4/2016	EPI_ISL_17767176	China	2016-Sep-01	Chinese Academy of Medical Sciences	Import from public-domain	Zhao, Z.
40	A/chicken/Guangdong/GD1602/2016	EPI_ISL_282397	China	2016-Mar-22	NA	Import from public domain	Sun, W.
41	A/chicken/Anhui/MZ33/2016	EPI_ISL_297930	China	2016-Feb-01	NA	Import from public domain	Liu, K.; Gu, M.; Gao, R.; Li, J.; Liu, D.; Sun, W.; Hu, J.; Xu, X.; Wang, X.; Liu, X.
42	A/black swan/Akita/1/2016	EPI_ISL_243058	Japan	2016-Nov-19	NA	Import from public domain	Okamatsu, M.; Hiono, T.; Matsuno, K.; Kida, H.; Sakoda, Y.
43	A/whooper swan/Iwate/5/2016	EPI_ISL_17767791	Japan	2016-Dec-18	Graduate School of Veterinary Medicine, Hokkaido University	Import from public domain	Sakoda, Y.; Okamatsu, M.; Matsuno, K.
44	A/whooper swan/Niigata/13/2017	EPI_ISL_17767801	Japan	2017-Jan-27	Graduate School of Veterinary Medicine, Hokkaido University	Import from public domain	Sakoda, Y.; Okamatsu, M.; Matsuno, K.
45	A/coot/Iwate/13/2016	EPI_ISL_256512	Japan	2016-Dec-22	NA	Hokkaido University	NA
46	A/chicken/Korea/HN1/2016	EPI_ISL_239261	Korea, Republic of	2016-Nov-16	NA	Animal and Plant Quarantine Agency (APQA)	NA
47	A/duck/Korea/ES2/2016	EPI_ISL_239262	Korea, Republic of	2016-Nov-16	NA	Animal and Plant Quarantine Agency (APQA)	NA
48	A/turtledove/Wuhan/HKBJ43/2015	EPI_ISL_205140	China	2015-Jan-01	NA	Import from public domain	Chen, L.-J.; Lin, X.-D.; Guo, W.-P.; Tian, J.-H.; Zhang, Y.-Z.
49	A/duck/Wuhan/WHYF02/2015	EPI_ISL_205115	China	2015-Jan-01	NA	Import from public domain	Chen, L.-J.; Lin, X.-D.; Guo, W.-P.; Tian, J.-H.; Zhang, Y.-Z.
50	A/duck/Hunan/01.21 YYFQH006-O/2015	EPI_ISL_199079	China	2015-Jan-21	NA	Institute of Microbiology, Chinese	NA

No.	Isolate name	GISAID no.	Country	Collection date	Originating Laboratory	Submitting Laboratory	Authors
51	A/goose/Anhui/AH350/2015	EPI_ISL_255822	China	2015-Dec-19	NA	Wuhan Institute of Virology	NA
52	A/muscovy duck/Vietnam/ LBM810/2015	EPI_ISL_230756	Vietnam	2015-Nov-10	NA	Import from public domain	Soda, K.; Nguyen, K.H.; Le, Q.M.; Ito, T.; Maegaki, S.
53	A/chicken/Vietnam/HU4-42/2015	EPI_ISL_293989	Vietnam	2015-Nov-07	NA	Import from public domain	Sakoda, Y.; Okamatsu, M.; Matsuno, K.; Jizou, M.
54	A/Environment/Hunan/07767/2015	EPI_ISL_219816	China	2015-Jan-08	NA	WHO Chinese National Influenza Center	Wang, Dayan; Li, Xiaodan; Zou, Shumei; Zhang, Ye; Bo, Hong; Li, Xiyan; Chen, Wenbing; Yang, Lei; Shu, Yuelong
55	A/goose/Hunan/110/2014	EPI_ISL_255827	China	2014-Nov-13	NA	Wuhan Institute of Virology	NA
56	A/duck/Eastern China/S0908/2014	EPI_ISL_208838	China	2014-Sep-08	NA	Import from public domain	Sun, H.; Sun, Y.; Pu, J.; Liu, L.; Li, C.; Xu, G.; Qin, M.; Zhang, Y.; Zhao, H.; Wei, K.; Liu, J.
57	A/duck/Hunan/233/2014	EPI_ISL_255493	China	2014-Nov-13	NA	Wuhan Institute of Virology	NA
58	A/duck/Hunan/HN334/2015	EPI_ISL_255757	China	2015-Dec-18	NA	Wuhan Institute of Virology	NA
59	A/duck/Hunan/HN336/2015	EPI_ISL_255759	China	2015-Dec-18	NA	Wuhan Institute of Virology	NA
60	A/duck/Sichuan/NCXJ15/2014	EPI_ISL_179647	China	2014-Apr-27	NA	Import from public domain	NA
61	A/Environment/Guangdong/40113/2015	EPI_ISL_219808	China	2015-May-11	NA	WHO Chinese National Influenza Center	Wang, Dayan; Li, Xiaodan; Zou, Shumei; Zhang, Ye; Bo, Hong; Li, Xiyan; Chen, Wenbing; Yang, Lei; Shu, Yuelong
62	A/Jiangsu/1/2020	EPI_ISL_718266	China	2020-Nov-28	Nanjing Municipality Center for Disease Control and Prevention	Nanjing Municipality Center for Disease Control and Prevention	NA
63	A/Anhui/2021–00011/2020	EPI_ISL_1081370	China	2020-Dec-22	Anhui Provincial Center for Disease Control and Prevention	WHO Chinese National Influenza Center	NA
64	A/chicken/Ha Tinh/514VTC/2021	EPI_ISL_9009552	Vietnam	2021-Feb-04	State Research Center of Virology and Biotechnology (VECTOR)	State Research Center of Virology and Biotechnology (VECTOR)	NA
65	A/chicken/Laos/WKU-716/2021	EPI_ISL_18388372	Lao, People's Democratic Republic	2021-Apr-20	NA	NA	Duong, B.T.; Than, D.; Yeo, J.S.; Theppangna, W.; Park, H.
66	A/duck/Cambodia/e10T241C18/2020	EPI_ISL_13295138	Combodia	2020-Sep-12	Institut Pasteur du Cambodia	The University of Hong Kong	NA

No.	Isolate name	GISAID no.	Country	Collection date	Originating Laboratory	Submitting Laboratory	Authors
67	A/duck/Vietnam/QN6800/2020	EPI_ISL_17809492	Vietnam	2020-Dec-25	Icahn School of Medicine at Mount Sinai	Icahn School of Medicine at Mount Sinai	Lizheng Guan; Gabriele Neumann; Yoshihiro Kawaoka

*We gratefully acknowledge the authors and originating and submitting laboratories for the sequences from GISAID's EpiFlu™ database (https://www.gisaid.org) on which this research is based. NA, not applicable; WHO, World Health Organization.

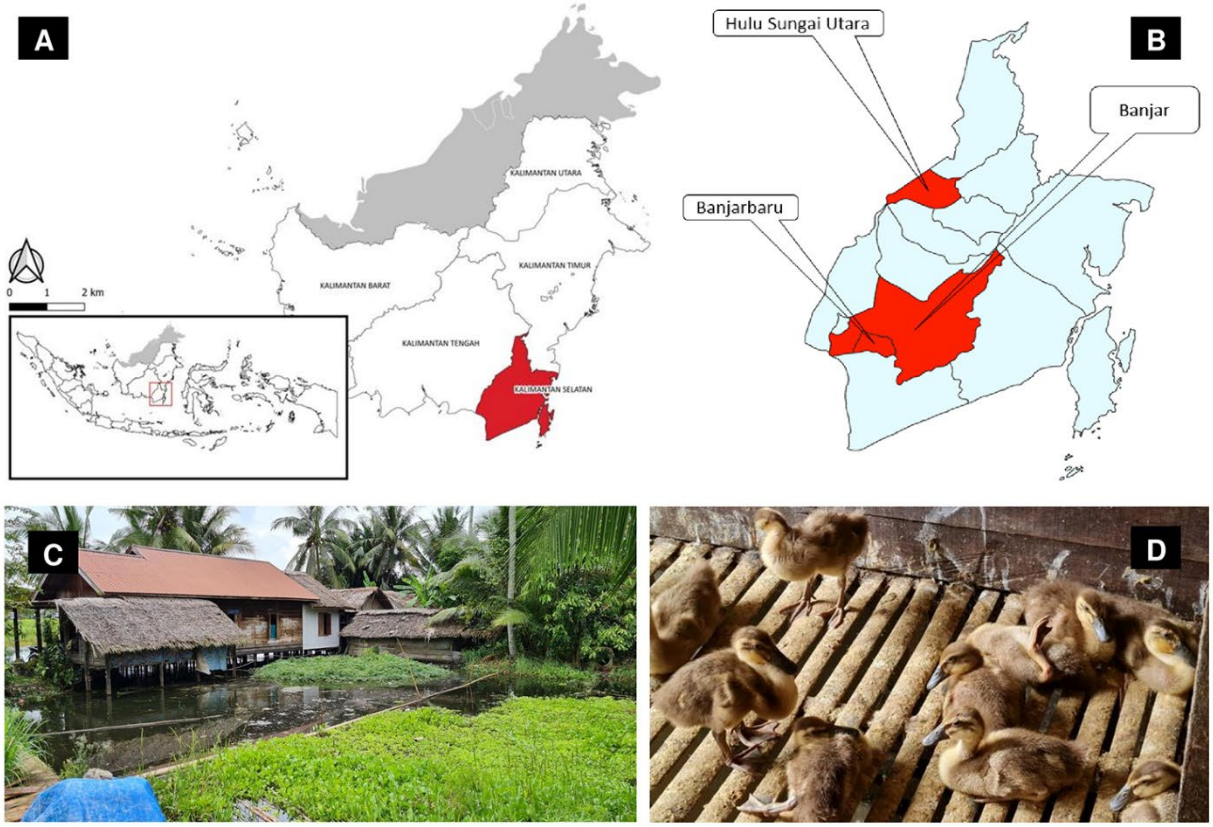

Appendix Figure 1. Duck farms in South Kalimantan Province, Indonesia, infected with highly pathogenic avian influenza A(H5N1) virus clade 2.3.4.4b. A) Map showing location of South Kalimantan Province, Indonesia (highlighted in red). Inset map shows the islands of Indonesia; red square indicates South Kalimantan Province. B) Map showing districts where H5N1 clade 2.3.4.4b–positive samples were collected from infected duck farms in Hulu Sungai Utara District (April 2022), in Banjarbaru District (July 2023), and from live bird markets in Banjar District (October 2022). C) Photo showing farms identified in the initial outbreak (April 2022) were located above a water flow from marshes. D) High mortality rate found in ducks infected with H5N1 clade 2.3.4.4b; younger ducks showed more severe disease with neurologic signs, such as torticollis and paralysis.

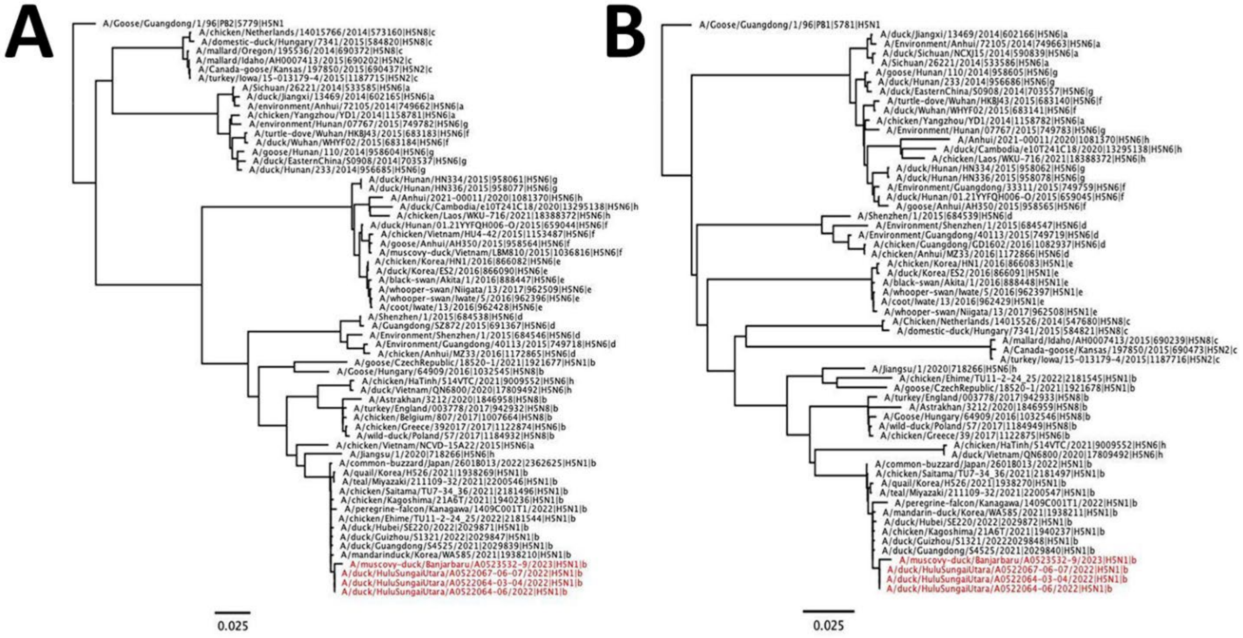

Appendix Figure 2. Phylogenetic analysis of polymerase basic 2 (A) and polymerase basic 1 (B) gene segments from highly pathogenic avian influenza A(H5N1) clade 2.3.4.4b viruses isolated during poultry outbreaks in South Kalimantan, Indonesia. Trees were constructed by using the maximum-likelihood method. Red font indicates viruses isolated from domestic ducks in this study compared with other H5 virus sequences from the GISAID database (https://www.gisaid.org). Scale bar indicates nucleotide substitutions per site.

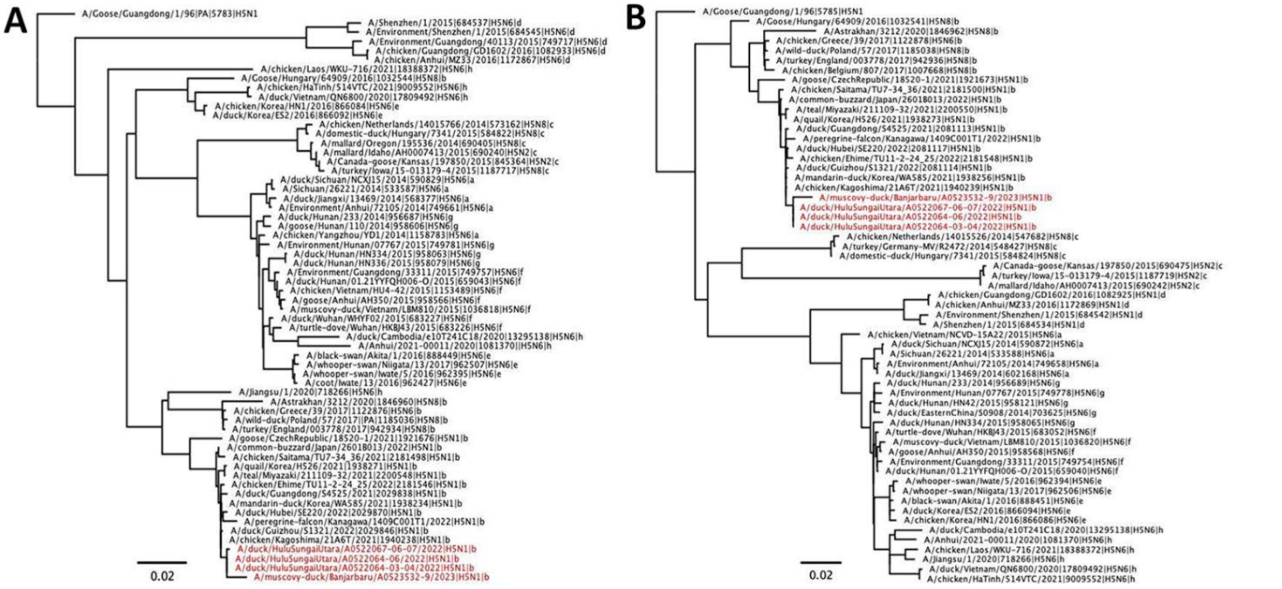

Appendix Figure 3. Phylogenetic analysis of polymerase acidic (A) and nucleoprotein (B) gene segments from highly pathogenic avian influenza A(H5N1) clade 2.3.4.4b viruses isolated during poultry

outbreaks in South Kalimantan, Indonesia. Trees were constructed by using the maximum-likelihood method. Red font indicates viruses isolated from domestic ducks in this study compared with other H5 virus sequences from the GISAID database (https://www.gisaid.org). Scale bar indicates nucleotide substitutions per site.

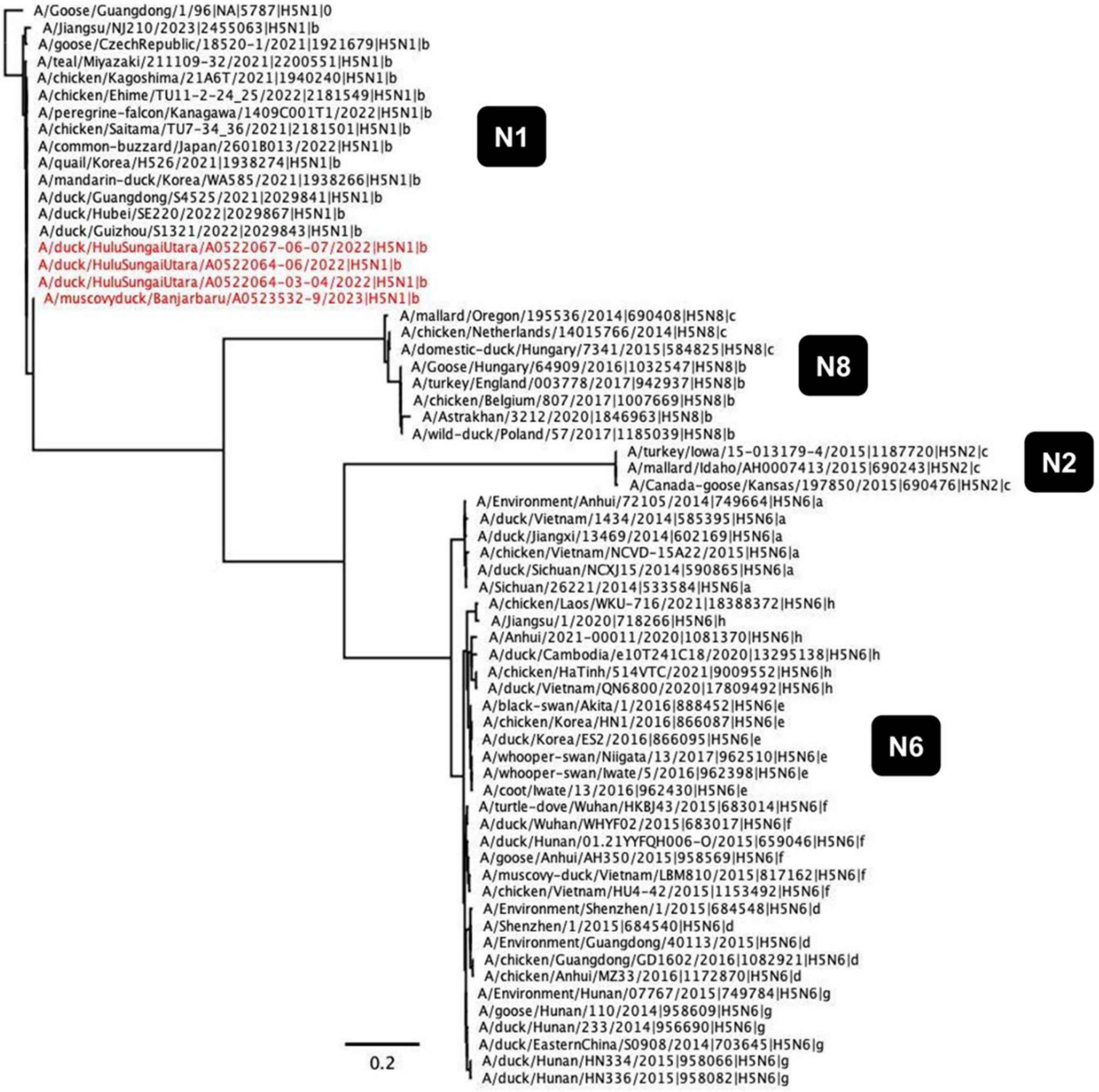

Appendix Figure 4. Phylogenetic analysis of neuraminidase gene segment from highly pathogenic avian influenza A(H5Nx) clade 2.3.4.4b viruses isolated during poultry outbreaks in South Kalimantan, Indonesia. Tree was constructed by using the maximum-likelihood method. Red font indicates viruses isolated from domestic ducks in this study compared with other virus sequences from the GISAID database (https://www.gisaid.org). Numbers in black boxes indicate viruses with neuraminidase subtypes N1, N2, N6, and N8. Scale bar indicates nucleotide substitutions per site.

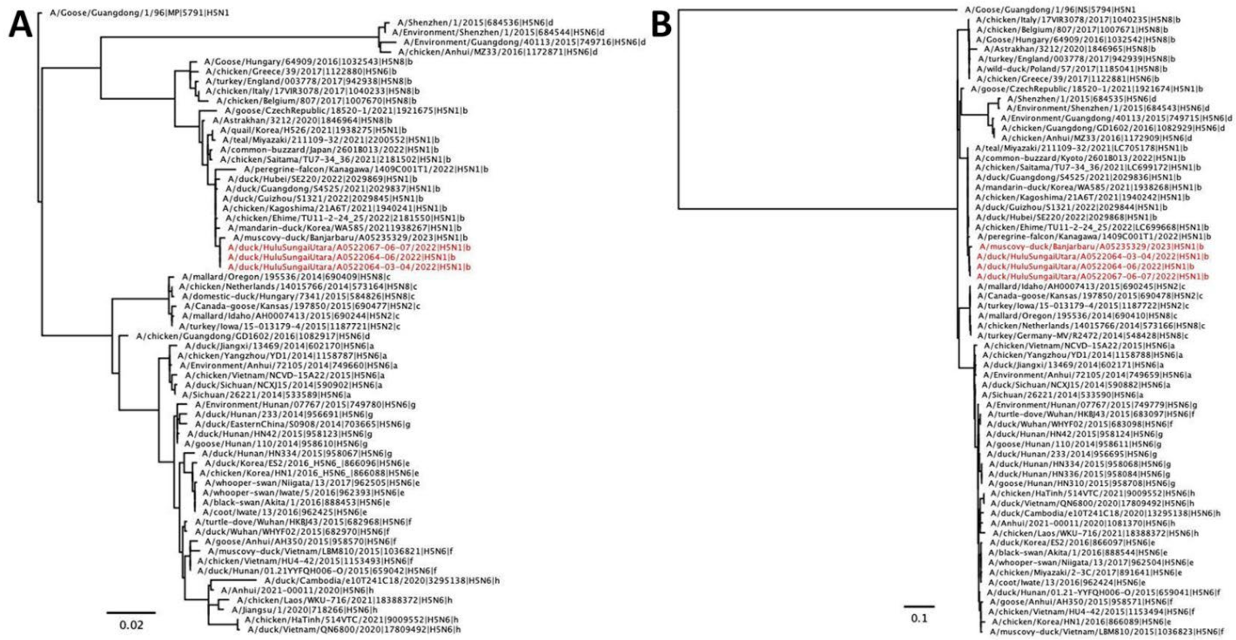

Appendix Figure 5. Phylogenetic analysis of matrix protein (A) and nonstructural protein (B) gene segments from highly pathogenic avian influenza A(H5N1) clade 2.3.4.4b viruses isolated during poultry outbreaks in South Kalimantan, Indonesia. Trees were constructed by using the maximum-likelihood method. Red font indicates viruses isolated from domestic ducks in this study, which were compared with other H5 virus sequences from the GISAID database (https://www.gisaid.org). Scale bar indicates nucleotide substitutions per site.

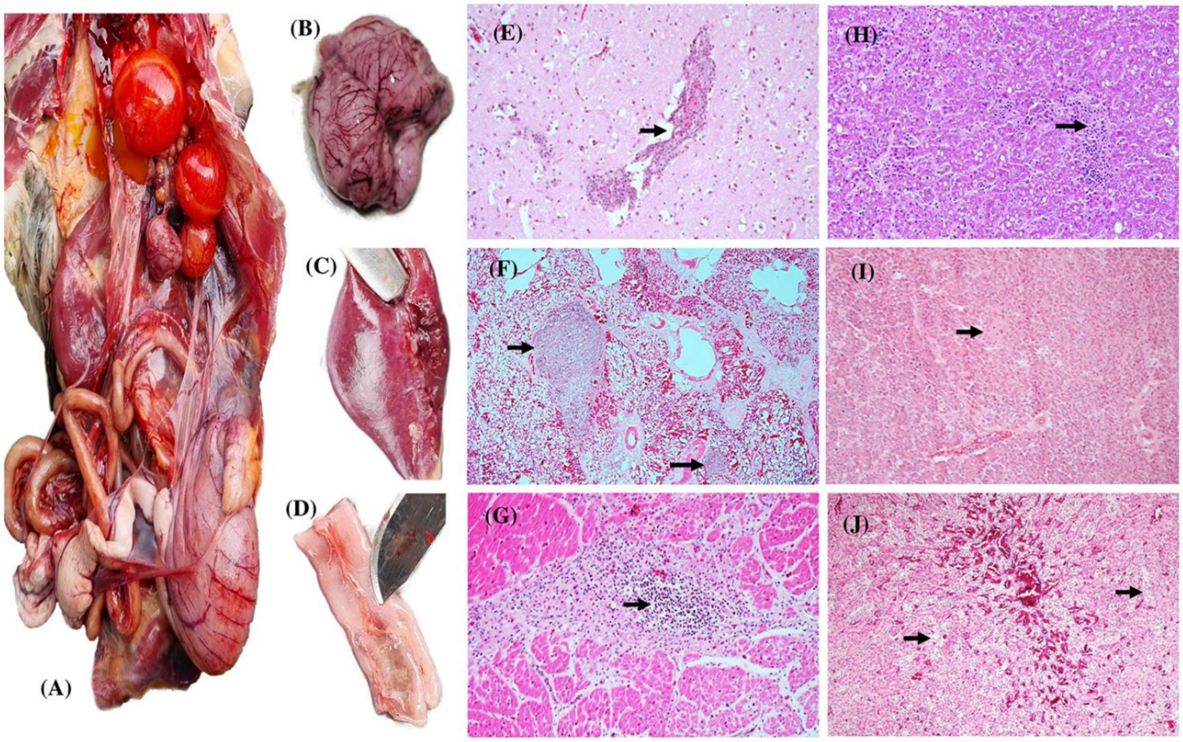

Appendix Figure 6. Gross and histologic pathology of ducks naturally infected with highly pathogenic avian influenza A(H5N1) clade 2.3.4.4b viruses in South Kalimantan, Indonesia in April 2022. A–D) Hemorrhages and acute necrosis observed in visceral organs (A) and in brain (B), spleen (C), and intestine (D). E–J) Tissue sections were stained with hematoxylin/eosin. E) Brain section shows congestion, edema, and vasculitis (arrow). F) Lung section shows congestion, perivascular edema, and lymphoid follicles (arrows). G) Heart section shows focal necrosis and inflammatory cell infiltrates (arrow). H) Liver section shows mild focal necrosis and inflammatory cell infiltrates (arrow) and lipid degeneration. I) Pancreas section shows hemorrhages and mild focal necrosis (arrow). J) Kidney section shows interstitial hemorrhages, congestion, and focal necrosis (arrow). E–J) Original magnification ×200.
